# Supplementary material for: Fast continuous streaming sort in big streaming data environment under fixed-size single storage
Source: PLoS One. 2022 Apr 5;17(4):e0266295. doi: 10.1371/journal.pone.0266295 (PMC8982863; doi:10.1371/journal.pone.0266295)
Supplement: Attachment — Submitted filename: Response to Reviewers.docx [file pone.0266295.s001.docx]

**ACADEMIC EDITOR**

- Many recent papers are missing.

**Author response:** We appreciate your concern and added recent papers in the introduction section of manuscript as follows:

- Systematic review and exploration of new avenues for sorting algorithm (2021).
- B*-sort: enabling write-once sorting for non-volatile memory (2020).
- Super sort sorting algorithm (2018).
- Permutations sorted by a finite and an infinite stack in series (2018).
- Montres : merge on-the-run external sorting algorithm for large data volumes on ssd based storage systems (2017).
- The limitations and drawbacks of the method should be highlighted.

**Author response:** We appreciate the academic editor for this comment and updated the manuscript. We added the discussion subsection in experimental results and discussion section on page 20.

Even though *fast streaming data sort* effectively sorts streaming data under the working memory limitation, there are two concerns about its limitation. First, the working memory size is 35% of the input data size. This means the proposed algorithm can reduce the working memory size up to 65%. On the other hand, the number of incoming data that can be handled by the proposed algorithm is up to $\frac{1}{0.35}=2.86$ times the working memory size without upgrading the memory space. Second, the proposed algorithm was designed for integer data, but it can be applied to sort floating-point numbers by multiplying each floating number with ${10}^{a}$, for an integer constant $a$. The value of $a$ must be predetermined from the maximum value of input data.

Journal Requirements:

1. Please ensure that your manuscript meets PLOS ONE's style requirements, including those for file naming. The PLOS ONE style templates can be found at 
<https://journals.plos.org/plosone/s/file?id=wjVg/PLOSOne_formatting_sample_main_body.pdf> and <https://journals.plos.org/plosone/s/file?id=ba62/PLOSOne_formatting_sample_title_authors_affiliations.pdf>

**Author response:** We appreciate your concern and have updated the manuscript. We improved the format of our manuscript according to the PLOS ONE style templates.

2. Please list all data sources used in your Data availability statement. Please also state whether or not sharing the Kaggle data with the manuscript complies with the terms and conditions of Kaggle.

**Author response:** We would like to thank you very much for this concern. We removed all data sources because the policy and the term of use of Kaggle in the fourth term. However, we cite all data source in the references section of the revised manuscript corresponding to the third term of use. Note that the terms of use can be found at
<https://www.kaggle.com/terms?fbclid=IwAR04PirD1LDMcqALlU3wAMkt-DHvf3ElUG8ZTNSXjhDLrSZGiCEV89CbOVY>.

3. We note that the grant information you provided in the ‘Funding Information’ and ‘Financial Disclosure’ sections do not match. When you resubmit, please ensure that you provide the correct grant numbers for the awards you received for your study in the ‘Funding Information’ section.

**Author response:** We would like to thank you very much for this concern. We updated the information as specified in cover letter.

4. Thank you for stating the following in the Acknowledgments Section of your manuscript: "This work was supported by Development and Promotion of Science and Technology Talents Project (DPST) and Thailand Research Fund under grant number RTA6080013." We note that you have provided funding information that is not currently declared in your Funding Statement. However, funding information should not appear in the Acknowledgments section or other areas of your manuscript. We will only publish funding information present in the Funding Statement section of the online submission form. Please remove any funding-related text from the manuscript and let us know how you would like to update your Funding Statement. Currently, your Funding Statement reads as follows: "Suluk Chaikhan received a grant from Development and Promotion of Science and Technology Talents Project (DPST). Chidchanok Lursinsap received a grant from Thailand Research Fund under grant number RTA6080013."

Please include your amended statements within your cover letter; we will change the online submission form on your behalf.

[Note: HTML markup is below. Please do not edit.]

**Author response:** We appreciate the reviewer for this comment and updated the manuscript. We removed all funding information in the acknowledgements section of the manuscript. Additionally, our amended statements are included within the cover letter.

**Reviewer 1**

1) The abstract must summarize the performance evaluation results.

**Author response:** We appreciate your concern and have revised the abstract. We added the comparison of sorting time with our proposed algorithm, bubble sort, quick sort, merge sort, *external merge sort*, and *streaming data sort* in the abstract. In addition, time and space complexities of the proposed algorithm were addressed in the abstract as well. The statements below were included in the revised abstract.

The algorithm was tested with various real data sets consisting of 10,000 to 17,000,000 numbers and different storage sizes ranging from 0.01n to 0.50n. It was found that the feasible lower bound of storage size is 0.35n with 100% sorting accuracy. The sorting time outperforms bubble sort, quick sort, insertion sort, and merge sort when data size is greater than 1,000,000 numbers. Remarkably, the sorting time of the proposed algorithm is 1,452 times less than the sorting time of *external merge sort* and 28.1767 times less than the sorting time of *streaming data sort*. The time complexity of proposed algorithm is $O(n)$ while the space complexity is $O(M)$.

2) The related work papers are not up to date in the domain where there are a lot of research papers are recently. In addition, the review for most of the cited work is nebulous. The review failed to summarize critical details about each of the cited research study and its relevant pros and cons.

**Author response:** We appreciate the reviewer for this comment and have updated the manuscript. We added recent papers and summarized the details of existing algorithms as suggested.

The following recent papers were added in the introduction section on pages 2 and 3.

- Systematic review and exploration of new avenues for sorting algorithm (2021).
- B*-sort: enabling write-once sorting for non-volatile memory (2020).
- Super sort sorting algorithm (2018).
- Permutations sorted by a finite and an infinite stack in series (2018).
- Montres : merge on-the-run external sorting algorithm for large data volumes on ssd based storage systems (2017).

The summary of the details of existing algorithms and *fast streaming data sort* was added to Table 1 of the introduction section on page 3.

Let $n$ be the size of streaming data, $M\ll n$ be the size of working memory, $k\geq1$, and $\epsilon>0$. The comparison of existing sorting algorithms and our *fast streaming data sort* are shown in Table 1, which contains eight columns: 1) compatibility with duplicate data, 2) stability, 3) compatibility with streaming data, 4) extra storage needed, 5) working space, 6) time complexity, 7) correct orders, and 8) correct values.

3) Text is overlapped with image in some figures.

**Author response:** We would like to thank you very much for this concern. The overlapped text on figures is caused by the pdf creation using latex with PLOS ONE format. The original figure can also be downloaded by the link named “Click here to access/download Figure;name.eps” on the topmost right of the figure. However, this problem was solved by rescaling the image to avoid the overlapped texts as shown below.

Edited

4) Equations should be aligned, and their fonts must be matched in the manuscript. All the equations should be revised and realigned.

**Author response:** We appreciate your concern and have updated the manuscript. According to your suggestion, all equations were revised and realigned on the left side, but the fonts were generated by LaTeX template of PLOS ONE.

5) The conclusions section should conclude that you have achieved from the study, contributions of the study to academics and practices, and recommendations of future works.

**Author response:** We appreciate the reviewer for this comment and have updated the manuscript accordingly. We added contributions of the study to academics and practices, and recommendations of future works to the conclusion section on pages 20 and 21.

The results were compared with several classic sorting algorithms and external merge sort on streaming data sort and analyzed with respect to large synthetic and real-world data sets. The sorting time of the proposed algorithm is 28.18 times less than streaming data sort. The proposed algorithm is faster than bubble sort and insertion sort when the size of input data is greater than 10,000 and faster than quick sort and merge sort when the size of input data is greater than 1,000,000. Moreover, the proposed algorithm is 1,452 times faster than external merge sort. Therefore, the proposed algorithm can achieve a remarkably faster sorting speed in comparison with the compared algorithms, even though memory overflow may occur in those classic sorting algorithms.

Theoretically, most of existing sorting algorithms cannot be applied to streaming data when working memory size is less than the size of incoming data. Although some algorithms are designed to cope with this situation, the sorting result is obtained with approximate orders and values. However, *fast streaming data sort* can sort streaming data and generate the sorting result with the exact order and values on the entire streaming sequence by using only 35% of data size as the size of available working memory. In the other hand, if the memory capacity is limited, *fast streaming data sort* is robust to overflow issue caused by increasing incoming data rather than other existing algorithms. In practical point of view, *fast streaming data sort* can be applied to several real-world applications as follows.

1. Reducing the searching time of enormous input data by using search algorithm on the sorting result in compact group form.
2. Finding order statistics, quartile, decile, and percentile of huge streaming data in social network applications on mobile phones, data cleaning, or data querying.
3. Examining duplicate data to reduce cost of storing data, to find error detection of systems, or to delete data in the data cleaning process.

Although *fast streaming data sort* can cope with the duplicate streaming data under the limitation of working memory less than the size of input data, there are two issues to be considered in the future work as follows.

1. Adding or improving new patterns of compact sequence to generate new compact group types that can be adopted to all different consecutive numbers.
2. Developing a compression procedure for compact groups with several data types, for instance, floating point number or text string, resulting in compatibility of data in many areas such as business, forecasting, logistics, and science.
6) The list of references should be reformatted and checked again to be matched with the journal requirement where a different styles and types are used. Please check some spells and typos.

**Author response:** We appreciate the reviewer for this comment and have updated the manuscript accordingly. We revised references based on PLOS ONE format and re-examine typos in the manuscript.

7) The authors should make their manuscript proofread by a native English speaker.

**Author response:** We would like to thank you very much for this concern. The manuscript was edited by [American Journal Experts](https://www.aje.com/) (AJE) and the editing certificate has been attached below.

**Reviewer 2**

1) The contribution is not stated clearly.

**Author response:** We appreciate the reviewer for this comment and have updated the manuscript accordingly. The contribution of the proposed algorithm was stated in abstract and conclusion section.

The statements below were included in the rewritten abstract section.

Big streaming data environment concerns a complicated scenario where data to be processed continuously flow into a processing unit and certainly cause a memory overflow problem. This obstructs the adaptation of deploying all existing classic sorting algorithms because the data to be sorted must be entirely stored inside the fixed-size storage including the space in internal and external storage devices. Generally, it is always assumed that the size of each data chunk is not larger than the size of storage ($M$) but in fact the size of the entire stream ($n$) is usually much larger than $M$. In this paper, a new fast continuous streaming sorting is proposed to cope with the constraint of storage overflow. The algorithm was tested with various real data sets consisting of 100,000 to 17,000,000 numbers and different storage sizes ranging from $0.01n$ to $0.50n$. It was found that the feasible lower bound of storage size is $0.35n$ with 100% sorting accuracy.

In the conclusion section, we added these statements on pages 20 and 21.

The results were compared with several classic sorting algorithms and external merge sort on streaming data sort and analyzed with respect to large synthetic and real-world data sets. The sorting time of the proposed algorithm is 28.18 times less than streaming data sort. The proposed algorithm is faster than bubble sort and insertion sort when the size of input data is greater than 10,000 and faster than quick sort and merge sort when the size of input data is greater than 1,000,000. Moreover, the proposed algorithm is 1,452 times faster than external merge sort. Therefore, the proposed algorithm can achieve a remarkably faster sorting speed in comparison with the compared algorithms, even though memory overflow may occur in those classic sorting algorithms.

Theoretically, most of existing sorting algorithms cannot be applied to streaming data when working memory size is less than the size of incoming data. Although some algorithms are designed to cope with this situation, the sorting result is obtained with approximate order and values. However, *fast streaming data sort* can sort streaming data and generate the sorting result with the exact order and values on the entire streaming sequence by using only 35% of data size as the size of available working memory. In the other hand, if the memory capacity is limited, *fast streaming data sort* is robust to overflow issue caused by increasing incoming data rather than other existing algorithms. In practical point of view, *fast streaming data sort* can be applied to several real-world applications as follows.

1. Reducing the searching time of enormous input data by using search algorithm on the sorting result in compact group form.
2. Finding order statistics, quartile, decile, and percentile of huge streaming data in social network applications on mobile phones, data cleaning, or data querying.
3. Examining duplicate data to reduce cost of storing data, to find error detection of systems, or to delete data in the data cleaning process.
2) The abstract must be rewritten, please delete unnecessary sentences.

**Author response:** We appreciate the reviewer for this comment and have rewritten the abstract accordingly. The new abstract has been given as shown below.

**Abstract**

Big streaming data environment concerns a complicated scenario where data to be processed continuously flow into a processing unit and certainly cause a memory overflow problem. This obstructs the adaptation of deploying all existing classic sorting algorithms because the data to be sorted must be entirely stored inside the fixed-size storage including the space in internal and external storage devices. Generally, it is always assumed that the size of each data chunk is not larger than the size of storage ($M$) but in fact the size of the entire stream ($n$) is usually much larger than $M$. In this paper, a new fast continuous streaming sorting is proposed to cope with the constraint of storage overflow. The algorithm was tested with various real data sets consisting of 100,000 to 17,000,000 numbers and different storage sizes ranging from $0.01n$ to $0.50n$. It was found that the feasible lower bound of storage size is $0.35n$ with 100% sorting accuracy. The sorting time outperforms bubble sort, quick sort, insertion sort, and merge sort when data size is greater than 1,000,000 numbers. Remarkably, the sorting time of the proposed algorithm is 1,452 times less than the sorting time of *external merge sort* and 28.1767 times less than the sorting time of *streaming data sort*. The time complexity of proposed algorithm is $O(n)$ while the space complexity is $O(M)$.

3) It is recommended to re-examine and design the Figures.

**Author response:** We appreciate your concern and have updated the manuscript. The overlapped text on figures is caused by the pdf creation using latex with PLOS ONE format. The original figure can also be downloaded by the link named “Click here to access/download Figure;name.eps” on the topmost right of the figure. However, this problem was solved by rescaling the image to avoid the overlapped texts as shown below.

Edited

4) A deep and detailed comparison with other methods is mandatory.

**Author response:** We would like to thank you very much for this concern and have updated the manuscript. We summarized the details of comparison of *fast streaming data sort* and existing algorithms in terms of compatibility with duplicate data, stability, compatibility with streaming data, extra storage needed, working space, time complexity, correct orders, and correct values in Table 1 of the introduction section on page 3.

Let $n$ be the size of streaming data, $M\ll n$ be the size of working memory, $k\geq1$, and $\epsilon>0$. The comparison of existing sorting algorithms and our *fast streaming data sort* are shown in Table 1, which contains eight columns: 1) compatibility with duplicate data, 2) stability, 3) compatibility with streaming data, 4) extra storage needed, 5) working space, 6) time complexity, 7) correct orders, and 8) correct values.

5) Please add the system specifications used for the evaluation as well as the programming language.

**Author response:** We would like to thank you for your valuable opinion. We added the system specifications to the first paragraph of the experimental results and discussion section on page 14.

The proposed algorithm was implemented by MATLAB R2021a and run on 3.2 GHz Intel Core i5 6500 and 8 GB of 2133 MHz RAM with the 64 bit Windows 10 platform.

6) Specify the limitations and drawbacks of the proposed method.

**Author response:** We appreciate your concern and have updated the manuscript. We added the discussion subsection to describe the limitations and drawbacks of the proposed method on page 20.

Even though *fast streaming data sort* effectively sorts streaming data under the working memory limitation, there are two concerns about its limitation. First, the working memory size is 35% of the input data size. This means the proposed algorithm can reduce the working memory size up to 65%. On the other hand, the number of incoming data that can be handled by the proposed algorithm is up to $\frac{1}{0.35}=2.86$ times the working memory size without upgrading the memory space. Second, the proposed algorithm was designed for integer data, but it can be applied to sort floating-point numbers by multiplying each floating number with ${10}^{a}$, for an integer constant $a$. The value of $a$ must be predetermined from the maximum value of input data.

7) Please add future work to the conclusion section and discuss it briefly.

**Author response:** We would like to thank you very much for this concern. We added future work at the end of conclusion section on page 21.

Although *fast streaming data sort* can cope with the duplicate streaming data under the limitation of working memory less than the size of input data, there are two issues to be considered in the future work as follows.

1. Adding or improving new patterns of compact sequence to generate new compact group types that can be adopted to all different consecutive numbers.
2. Developing a compression procedure for compact groups with several data types, for instance, floating point number or text string, resulting in compatibility of data in many areas such as business, forecasting, logistics, and science.
